# A Rare Case of Acute Pancreatitis Secondary to Marijuana Use: A Case Report

**DOI:** 10.7759/cureus.65882

**Published:** 2024-07-31

**Authors:** Hemangi Patel, Nithya Devanathan, Pierr Bojaxhi, Alejandro Biglione

**Affiliations:** 1 Sports Medicine, Nova Southeastern University Dr. Kiran C. Patel College of Osteopathic Medicine, Fort Lauderdale, USA; 2 Medicine, Nova Southeastern University Dr. Kiran C. Patel College of Osteopathic Medicine, Fort Lauderdale, USA; 3 Internal Medicine, Wellington Regional Medical Center, Wellington, USA

**Keywords:** pancreatitis causes, abdominal pain in females, substance abuse, marijuana use, acute pancreatitis

## Abstract

The prevalence of cannabis use for recreational and medicinal purposes has steadily increased. While it is commonly used to alleviate pain, its use is also associated with many acute and chronic adverse effects. There are cases reported on the negative impact of cannabis use on gastrointestinal (GI) disorders; however, there have been few reported cases linking cannabis use to acute pancreatitis. This case report discusses a 37-year-old female presenting to the emergency department for cannabis-induced acute pancreatitis. The purpose of this case report is to educate on the importance of recognizing the potential GI complications resulting from marijuana use.

## Introduction

Cannabis is used by 147 million people worldwide, predominantly in the 18- to 25-year-old population [1]. It is mostly used for recreational purposes and medically for the management of pain and inflammation and treatment of neurological diseases [2]. There can be many adverse effects associated with marijuana use, such as hyperemesis syndrome, cognition decline, cardiovascular events such as myocardial infarctions, and psychiatric symptoms [3]. Furthermore, there is research that shows marijuana influences the gastrointestinal (GI) system function as well [3]. Recent studies have found a correlation between the incidence of acute pancreatitis in individuals consuming cannabis [1]. 

The proposed mechanism of cannabis on the GI system involves cannabinoid receptors (CB) 1 and 2. CB1 receptors are found in the central nervous system, colonic epithelium, and submucosa of the myenteric plexus [4]. Activation of these receptors can lead to an increase in appetite, a decrease in pain perception, and alterations to motility, contractility, and secretions of GI contents. CB2 receptors play a role in the anti-inflammatory processes and decrease GI motility [4]. The interplay between these two receptors is modulated by the use of marijuana, leading to an overactivation or desensitization with chronic use [5]. CB1 and CB2 are extensively found in the pancreas, but their role in the pathogenesis of pancreatitis is not well understood [4]. There are a few reports that discuss acute pancreatitis in cannabis use similar to the case presented. It is hypothesized that the CB receptors affect the sphincter of Oddi, inducing pancreatitis [1,2,6]. 

The classic presentation of acute pancreatitis includes epigastric pain, nausea, vomiting, and fevers. It is diagnosed when a patient presents with two out of the three findings: abdominal pain suggestive of pancreatitis, elevated levels of amylase and/or lipase greater than three times the normal level are specific for acute pancreatitis, findings of enlargement of the pancreas and/or irregular contour of the pancreas, and/or the presence of peripancreatic fat stranding on diagnostic imaging [6,7]. It is most frequently caused by alcohol abuse, gallstones, hypertriglyceridemia, medications, infection, malignancy, and recreational drugs [6]. The incidence of cannabis-induced pancreatitis is lower than 2% in the general population [6] and higher in individuals less than 35 years of age [1]. 

With the gradual reduction of restrictions on cannabis access and usage, the potential for medical complications associated with cannabis use is becoming increasingly apparent. We report the case of a 37-year-old female presenting to the emergency department with abdominal pain and was eventually found to have cannabis-induced acute pancreatitis.

## Case presentation

This is a 37-year-old female who presented to the emergency room with abdominal pain that was located in the epigastric and left upper quadrant pain for four days. She described the pain as intermittent, ranging from dull to sharp and radiating to the back. She denied any chest pain or radiation of pain to the shoulders. She also had associated nausea and vomiting with worsening intensity after ingesting food. She had never experienced this pain before. Her past medical history included anxiety and depression. She had no other significant past medical history, including any respiratory disorders such as bronchitis. She denied a past surgical history. She did not have a family history of pancreatitis. She was previously prescribed a selective serotonin reuptake inhibitor (SSRI) medication two years ago to help with her anxiety and depression, but she was unable to identify the medication name or dosage. She stopped the medication because smoking medical marijuana controlled her anxiety and depression better, and she did not like the concept of using psychiatric medications. The level of control was significant with medical marijuana, and she described it as being able to perform day-to-day activities without any significant symptoms of anxiety or depression. She had a history of recreational marijuana use since she was a teenager, which improved her anxiety and depression at that time. Her desire to avoid psychiatric medications led her to use medical marijuana currently instead of solely for recreational use.

She denied alcohol use or tobacco use. She reported a consistent pattern of marijuana use, smoking ¼ oz of marijuana two to three times daily over the course of the past 12 months to control her symptoms of anxiety and depression. The exact strain of cannabis was unknown, and if any filter was used during the consumption of marijuana by smoking was unknown. In the emergency room, her vital signs showed a temperature of 98.2°F (36.8°C), a heart rate of 93 beats per minute, a blood pressure of 102/68 mmHg, and a respiration rate of 20 breaths per minute. Her oxygen saturation was 97% on room air. Her BMI was 21.61 kg/m^2^, which is within normal limits. On physical examination, heart sounds were normal S1 and S2, no S3 or S4, lungs were clear to auscultation, and an abdominal exam revealed no abnormalities on inspection. On palpation, there was epigastric and left upper quadrant tenderness. On auscultation, the abdomen revealed hypoactive bowel sounds.

The patient's admission labs are displayed in Table 1. 

**Table 1:** Normal lab value range compared to the patient's lab values on admission Na: sodium; K: potassium; Cl: chloride; CO_2_: carbon dioxide; BUN: blood urea nitrogen; AST: aspartate aminotransferase; ALT: alanine transaminase; WBC: white blood cells; RBC: red blood cells; HDL: high-density lipoprotein

	Patient Value	Normal Range
Na	135 mmol/L	135-145 mmol/L
K	4.0 mmol/L	3.4-4.5 mmol/L
Cl	106 mmol/L	95-108 mmol/L
CO_2_	27 mEq/L	23-28 mEq/L
BUN	13 mg/dL	8-21 mg/dL
Creatinine	0.86 mg/dL	0.8-1.3 mg/dL
AST	15 U/L	5-30 U/L
ALT	20 U/L	5-30 U/L
Alkaline phosphatase	50 U/L	50-100 U/L
Bilirubin	0.5 mg/dL	0.3-1.2 mg/dL
WBC	8.57 x 10^3^/mcL	4.5-11 x 10^3^ cells/mcL
RBC	4.44 x10^6^/mcL	4.2-5.9 x 10^6^ cells/mcL
Hemoglobin	13.5 g/dL	12-16 g/dL (females) 14-17 g/dL (males)
Hematocrit	40.30%	36-46% (females) 41-53% (males)
Platelets	194 x10^3^ cells/mcL	150 x10^3^ cells/mcL - 450 x 10^3^ cells/mcL
Lipase	5,221 IU/L	0-160 IU/L
Cholesterol	204 mg/dL	Less than 200 mg/dL
Triglycerides	79 mg/dL	Less than 150 mg/dL
HDL	90 mg/dL	Above 40 mg/dL

**Figure 1:**
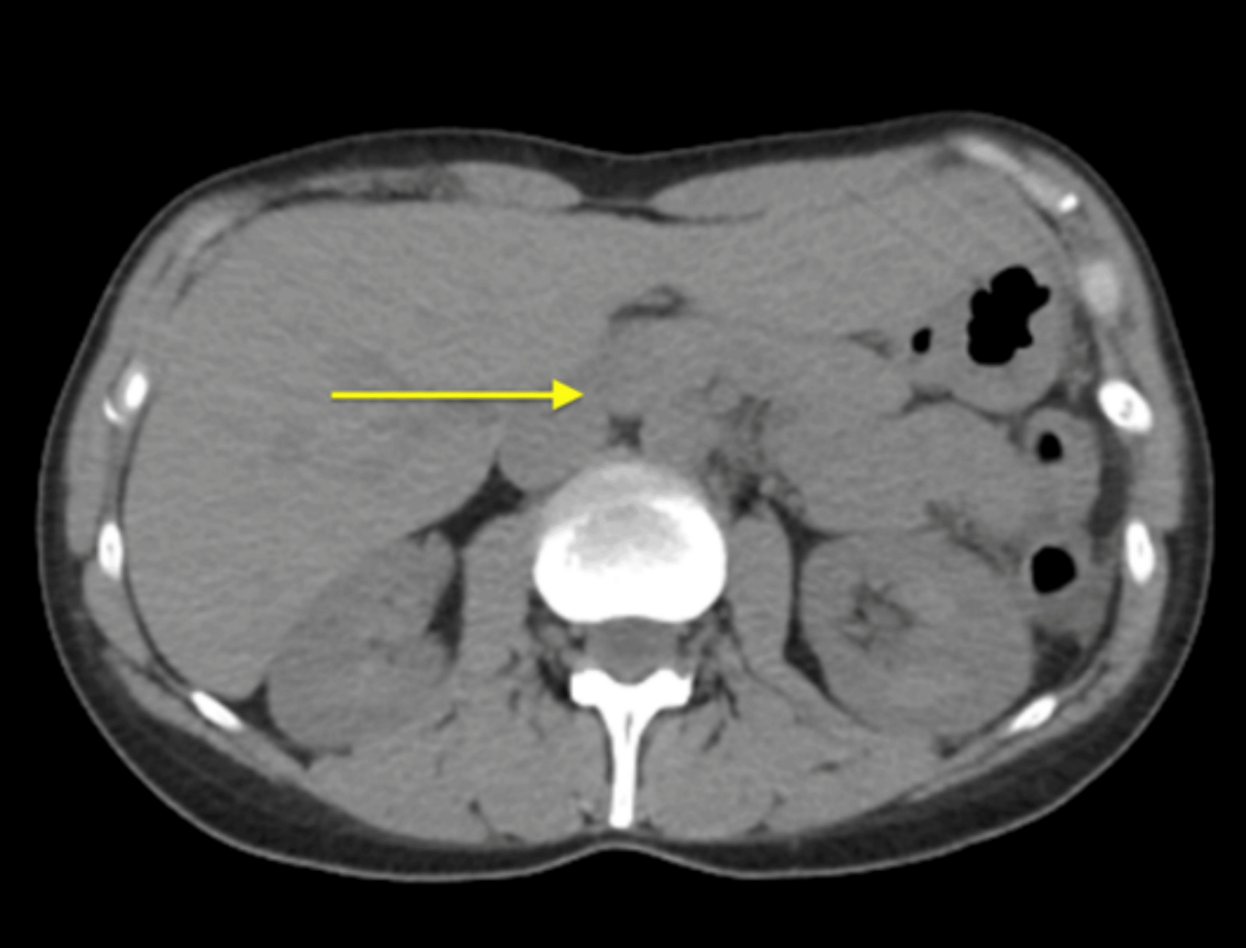
Computerized tomography (CT) abdomen/pelvis without contrast shows mild fat stranding present in the region of the pancreas consistent with acute pancreatitis.

**Figure 2:**
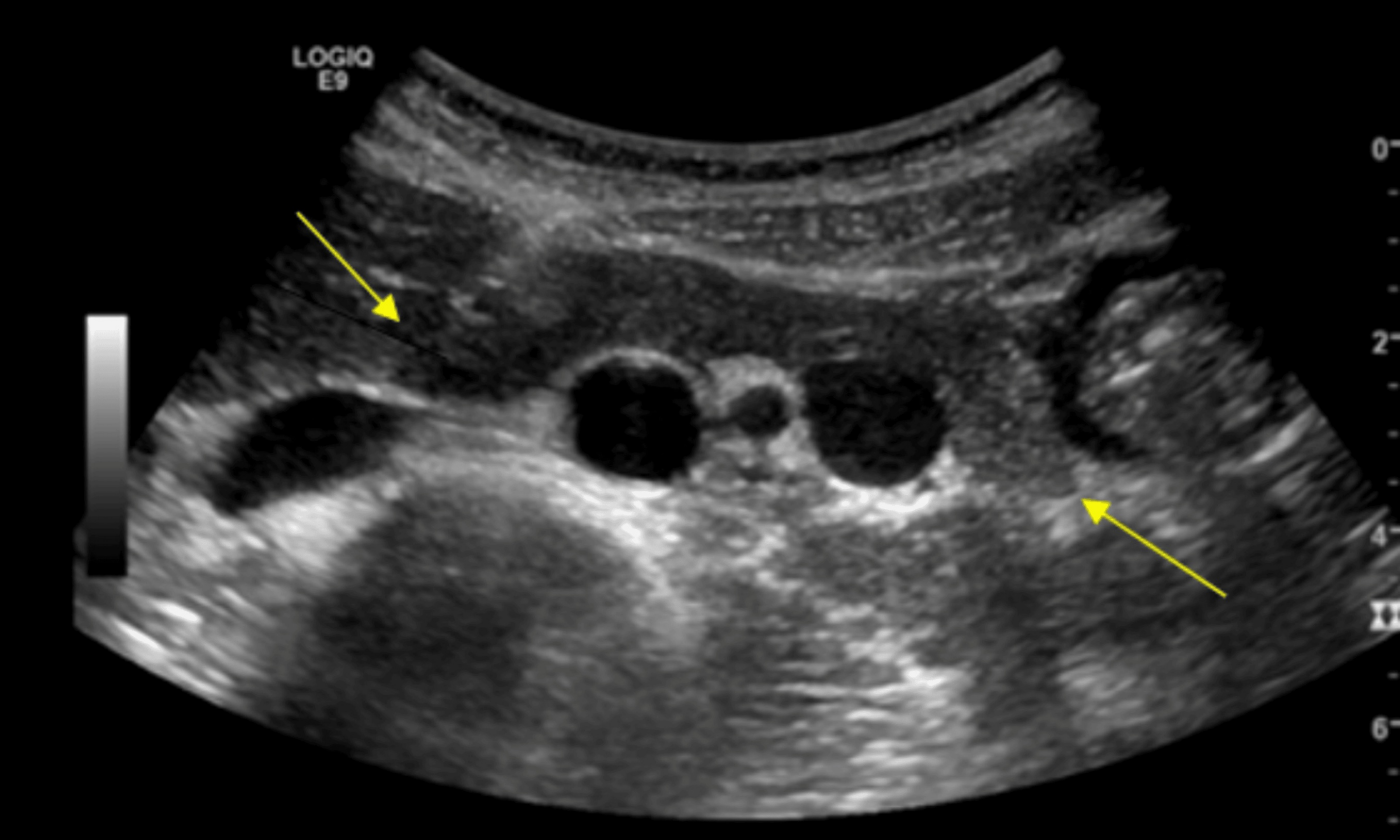
Ultrasound of the abdomen shows the pancreas is homogeneously enlarged with a mildly hypoechogenic appearance.

**Figure 3:**
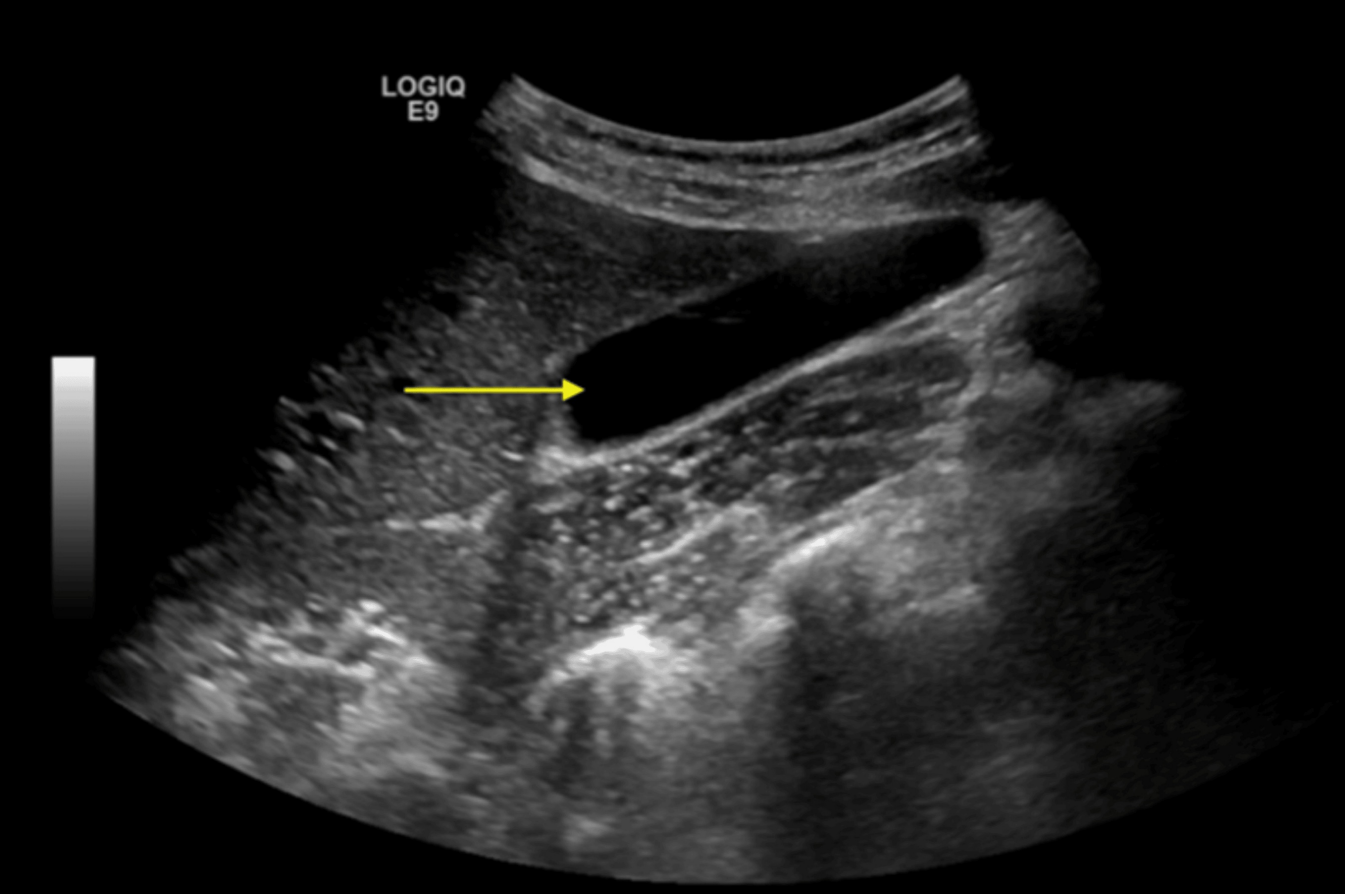
Ultrasound of the right upper quadrant (RUQ) shows a normal-appearing gallbladder without gallstones.

**Figure 4:**
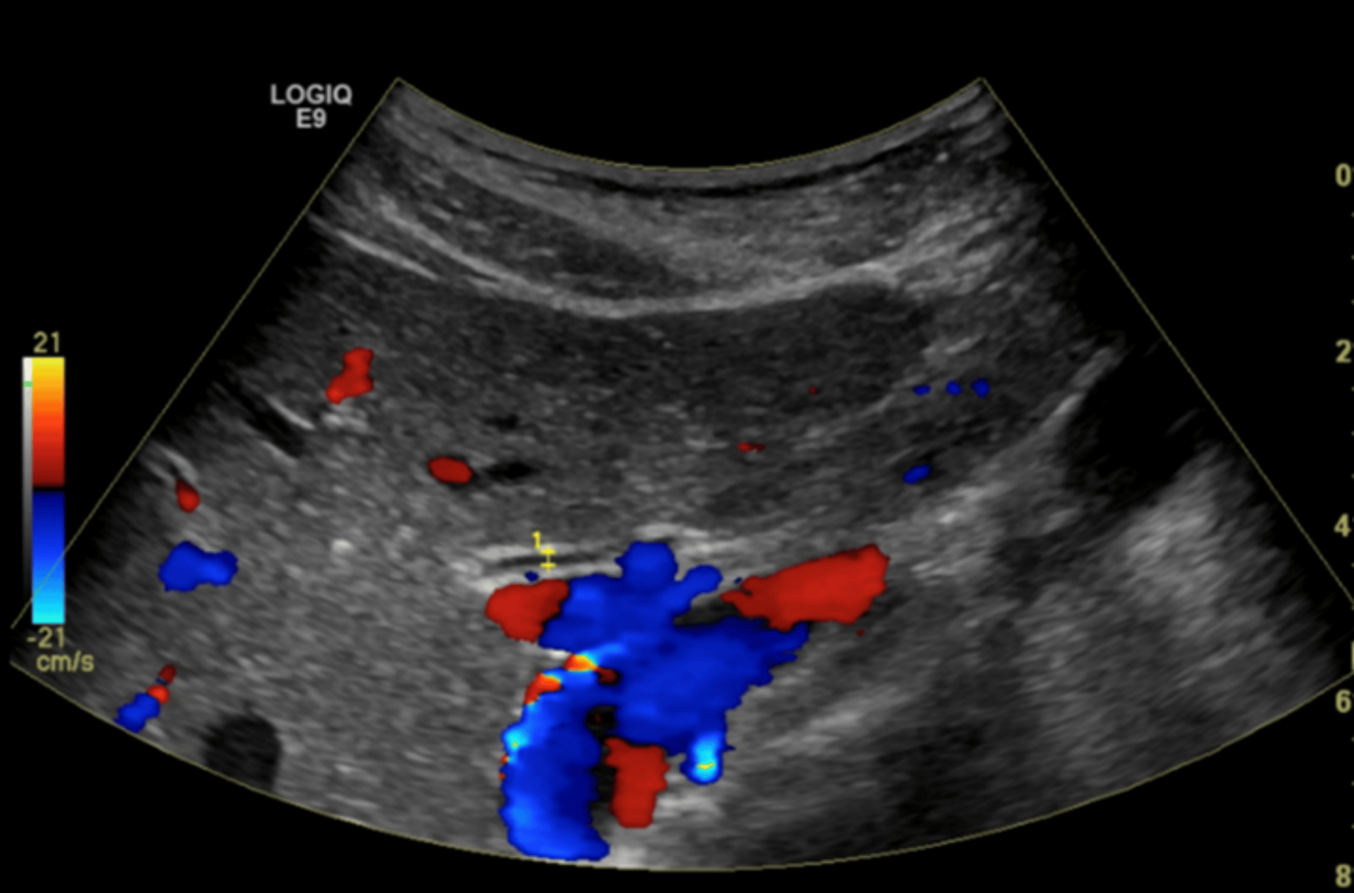
Ultrasound of the right upper quadrant (RUQ) reveals no dilation to the common bile duct, which measures 5 mm.

**Figure 5:**
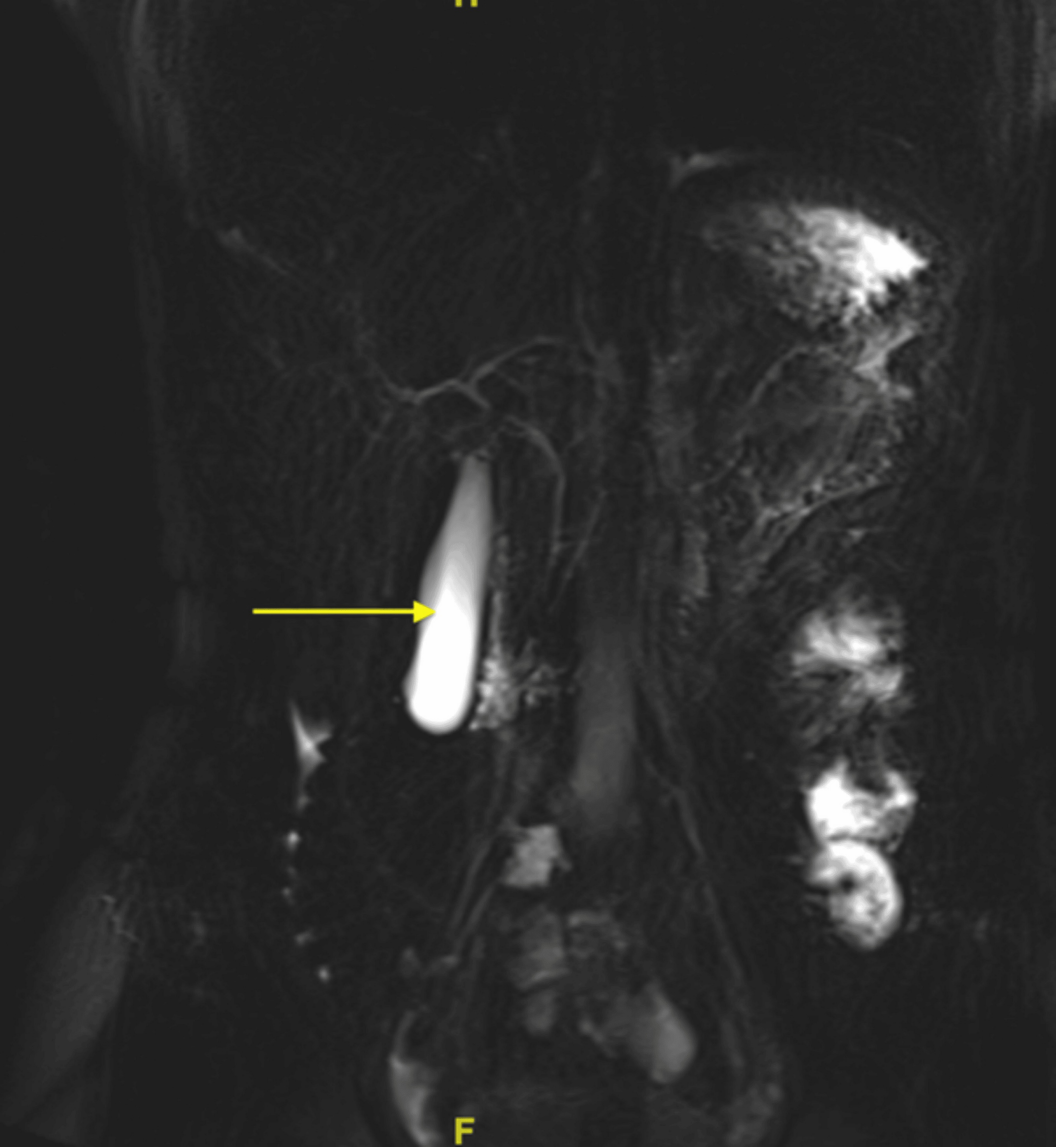
Magnetic resonance cholangiopancreatography (MRCP) reveals the right and left common biliary ducts without abnormalities. There is a distended gallbladder present with no thickening.

Her chest x-ray (CXR) did not reveal any abnormalities. The computed tomography (CT) of the abdomen and pelvis without contrast revealed the normal size of the liver, a normal appearance of the gallbladder, and a mildly enlarged pancreas surrounded by mild fat stranding (Figure 1). The spleen, kidneys, and appendix were within normal limits; sigmoid diverticulosis was present, but there was no evidence of bowel obstruction. The bladder and uterus findings were unremarkable. An ultrasound of the right upper quadrant showed no liver masses or dilated intrahepatic biliary ducts present and no apparent cyst or mass of the pancreas (Figure 2). The gallbladder demonstrated no gallstones or wall thickening, with no sonographic Murphy’s sign (Figure 3). The common bile duct measured 5 mm in diameter (Figure 4). The spleen, kidneys, adrenal glands, and gallbladder were unremarkable. There were no abdominal masses or fluid collection present. Magnetic resonance cholangiopancreatography (MRCP) showed a normal common bile duct of 5 mm and a distended gallbladder with no thickening (Figure 5). 

The patient was admitted for management of acute pancreatitis and treated with lactated ringers 1,000 mL solution intravenous (IV) at 150 mL/hour, ondansetron 4 mg for nausea and vomiting as needed, and toradol 15 mg IV push one time at the initial encounter for pain management. Throughout her admission, she was given morphine 2 mg IV push as needed for pain, acetaminophen 1,000 mg IV every six hours for pain, and ketorolac 10 mg every six hours for pain interchangeably. Her symptoms resolved, and she was discharged two days later with a follow-up with her primary care physician. She was advised to abstain from smoking marijuana. 

## Discussion

Cannabis is one of the most commonly used recreational drugs in the United States. It is often sought out for its therapeutic effects on anxiety, chronic pain, epilepsy, irritable bowel syndromes, and other health conditions [1,8]. Despite these beneficial effects, the general population is mostly unaware of the potential harmful effects that come with chronic use. Some of the neurological implications of marijuana include cognitive impairment, psychoactive impairment, altered brain development, and an increased risk of psychosis disorders such as schizophrenia [1,9]. Similarly, cannabis use can cause disruption to the GI system, leading to complications such as hyperemesis syndrome, biliary dyskinesia, and potentially being associated with acute pancreatitis [4].

Acute pancreatitis is commonly caused by alcohol, gallstones, and medications [2]. The diagnosis ranges from mild epigastric pain to severe organ dysfunction and varies in each individual [2]. The most common symptoms are abdominal pain, nausea, and vomiting. In the last few decades, there has been a gradual rise in the incidence of acute pancreatitis. It has been theorized that the increasing use of cannabis could be a contributing factor in the rise in acute pancreatitis diagnoses [2]. This case described a patient who presented with the classical clinical presentation of acute pancreatitis: abdominal pain, nausea, and vomiting.

Cannabis use should be highly considered in a patient who presents with acute pancreatitis and has a history of cannabis use [10,11]. In a recent systematic review by Barkin et al., 26 cases of cannabis-induced acute pancreatitis have been present. Once cannabis use was discontinued, more than half the cases had no recurrence of acute pancreatitis episodes. Additionally, chronic cannabis use potentially induces acute pancreatitis through the binding of tetrahydrocannabinol (THC) to the pancreatic CB1 and CB2 receptors, suggesting involvement of the endocannabinoid system in the pathogenesis [1]. This relationship further emphasizes the importance of proper history-taking and consideration of cannabis use when a patient presents with acute pancreatitis.

## Conclusions

Cannabis is commonly used for therapeutic purposes. The number of cases of cannabis-induced complications is steadily increasing. An example of a cannabis-induced complication is acute pancreatitis. We presented the case of a young female who developed acute pancreatitis secondary to cannabis use. The purpose of this case report is to increase awareness of the importance of proper history-taking, including the use of recreational drugs and medical marijuana use in cases of acute pancreatitis. This case report also emphasizes the importance of maintaining awareness regarding possible GI complications with cannabis use, including acute pancreatitis. This will result in avoiding a costly workup looking for unusual causes of pancreatitis and increasing patient awareness of the possible adverse effects of the recreational or medical use of cannabis.
